# Study on Retardation Factors of Cr(VI) Transport in Typical Soils of China

**DOI:** 10.3390/toxics13090774

**Published:** 2025-09-13

**Authors:** Xiongbiao Qiao, Xiangyang Zhang, Dejin Zhou, Ning Sun, Zhenyu Ding, Liping Bai, Zongwen Zhang

**Affiliations:** 1 State Key Laboratory of Soil Pollution Control and Safety, Chinese Academy of Environmental Planning, Beijing 100043, China; qiaoxb@caep.org.cn (X.Q.);; 2 School of Water Resources and Environment, China University of Geosciences, Beijing 100083, China; 2005230032@email.cugb.edu.cn; 3 Hainan Mining Co., Ltd., Changjiang 572700, China; 4 Technical Centre for Soil, Agriculture and Rural Ecology and Environment, Ministry of Ecology and Environment, Beijing 100012, China

**Keywords:** Cr(VI) transport, dispersion coefficient, groundwater, retardation factor, soil screening values

## Abstract

Chromium (VI) mobility in soils critically influenced groundwater contamination risks, but accurate predictions were hindered by the lack of systematic retardation factor (R) data across China’s diverse soils. This study combined Bromide anion (Br^−^)-tracer and Cr(VI) column leaching experiments with CXTFIT code to determine dispersion coefficients (D) and R values in seven representative Chinese soils (e.g., brown soil, black soil), with model validation against Br^−^ tracer data. By comparing deterministic equilibrium and two-region non-equilibrium models, the research demonstrated that the non-equilibrium approach better characterized Cr(VI) transport, revealing significant soil-dependent R variations (1.09–16.13). Particularly noteworthy was the exceptional Cr(VI) retention observed in Heilongjiang black soil (R > 10), which indicated strong immobilization capacity. As China’s first systematic Cr(VI) retardation database, these findings provided essential parameters for predicting Cr(VI) mobility at contaminated sites, refining risk assessment models, and designing soil-specific remediation strategies—particularly crucial for high-retention regions. Methodologically, this work established an integrated experimental-modeling framework that addressed soil heterogeneity, while its outcomes directly supported regulatory frameworks through updated soil screening values. These findings provided scientific support for formulating region-specific soil management policies, with particular implications for environmental protection and agricultural safety in Cr(VI)-contaminated black soil regions.

## 1. Introduction

Chromium Cr(VI) is a widespread contaminant in soil and groundwater, primarily originating from industrial waste discharges associated with metallurgy, metal processing, electroplating, and related industries [1]. Cr(VI) is highly bioavailable and can readily accumulate in human organs, posing significant health risks. While Cr(VI) plays essential biological roles in trace amounts, excessive Cr(VI) exposure may cause severe health effects, including respiratory diseases, gastrointestinal disorders, allergic contact dermatitis, and even cancer [2,3]. The World Health Organization (WHO) has set a stringent maximum contaminant level (MCL) of 0.05 mg/L for Cr(VI) in drinking water due to its well-documented toxicity. Given these serious health implications and its environmental mobility, Cr(VI) is classified as a high-priority hazardous pollutant [4]. China is the world’s largest producer of chromium chemicals and serves as a global hub for leather tanning and electroplating industries, accounting for over 50% of global production [5]. In recent years, the continuous adjustment of China’s industrial structure and the increasingly stringent environmental protection requirements have resulted in the closure of numerous chromium-related enterprises. This has led to the abandonment of a significant number of chromium-contaminated sites, which have the potential to become sources of Cr(VI) contamination [6]. Chromium concentrations in China’s agricultural soils exhibited distinct temporal variations, with average Cr levels increasing from 56.52 mg/kg (1989–2000) to 75.36 mg/kg (2006–2010), followed by a decline to 70.11 mg/kg (2011–2016). These fluctuations in farmland soils reflect the combined effects of industrial development and subsequent environmental regulations implemented during these periods [7,8].

Cr(VI) belongs to a class of highly mobile heavy metal ions. Under specific environmental conditions, it can undergo redox conversion to Cr(III) [9]. Following soil infiltration, Cr(VI) can migrate through soil matrices and disseminate via leachate into aquifers, leading to groundwater contamination at regional scales. The migration behavior of Cr(VI) is strongly influenced by the physicochemical properties of different soil types, which exhibit substantial variability [10,11]. In general, Cr(VI) undergoes a series of physicochemical and biological processes, including diffusion, convective dispersion, and sorption attenuation, within transport media [12]. As a result, Cr(VI) migration typically occurs at a reduced rate compared to the surrounding aqueous phase, demonstrating delayed transport behavior. The retardation factor (R) quantitatively characterizes this delayed migration, representing the combined effects of soil sorption capacity and physical barrier mechanisms on Cr(VI) mobility [13]. A higher retardation factor indicates a weaker migration ability of Cr(VI) within the medium [14]. The retardation capabilities of different soil types vary, and thus the migration abilities of the same contaminant may differ in different soil types, resulting in disparate pollution scenarios. It is therefore of the utmost importance to investigate the retardation factors of contaminants in a variety of soil types in order to protect the ecological environment, assess human health risks and calculate groundwater screening values for protection [15]. With growing concerns over environmental degradation, research on retardation factors has gained increasing attention. However, most existing studies have primarily focused on examining retardation effects for various soil contaminants [16,17]. To date, no studies have specifically investigated Cr(VI) retardation factors in typical Chinese soil systems. As a key parameter governing contaminant migration from soils to groundwater, the retardation factor significantly influences the environmental fate of Cr(VI) [18].

The primary objectives of this study are to (1) develop a robust methodology for determining retardation factors (R) and (2) systematically quantify Cr(VI) retardation across seven representative Chinese soil types. The derived R values will establish critical parameters for calculating region-specific soil screening values to protect groundwater resources nationwide. Importantly, the substantial variability in soil-specific retardation capacities directly influences contaminant migration rates and spatial pollution distribution patterns. Accurate determination of these factors is essential for advancing predictive models of Cr(VI) transport dynamics and optimizing site-specific risk management strategies.

## 2. Materials and Methods

### 2.1. Test Soils

**Figure 1:**
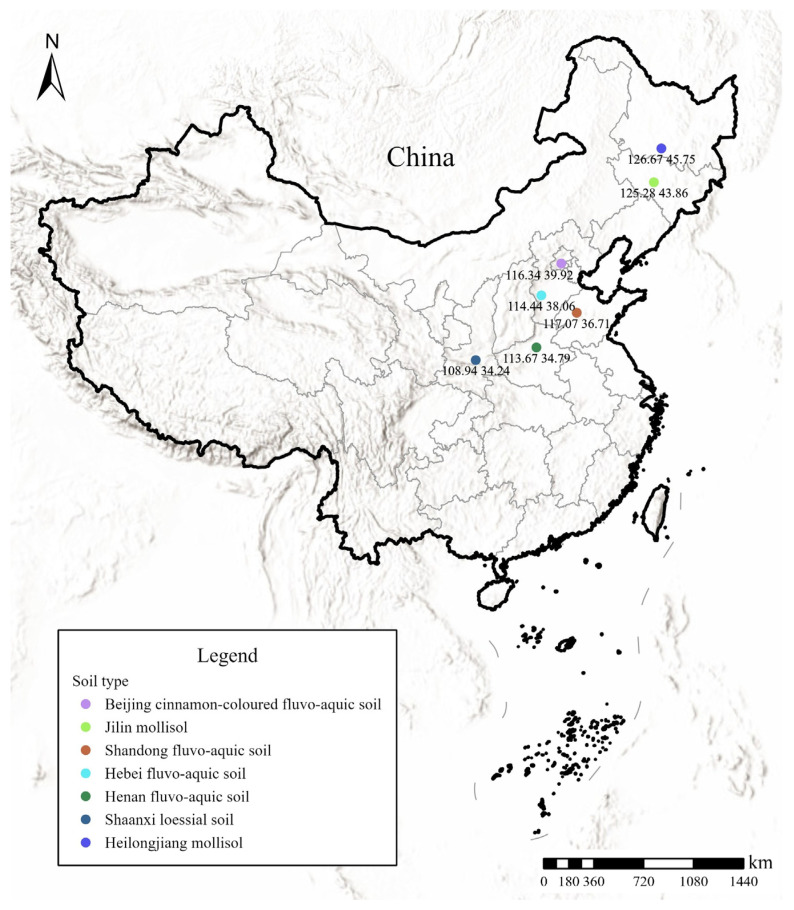
Sampling point locations and longitude–latitude coordinate diagram.

Representative soil samples were collected from major agricultural regions across China to encompass diverse geographical and pedological characteristics. Surface soils (0–20 cm depth) were obtained from scientific experimental stations in seven distinct locations: Beijing cinnamon-colored fluvo-aquic soil, Henan fluvo-aquic soil, Hebei fluvo-aquic soil, Shandong fluvo-aquic soil, Shaanxi loessial soil, Jilin mollisol, and Heilongjiang mollisol, and the samples were collected during June to July 2016 (summer season), with the geographical locations of the sampling sites shown in Figure 1. The properties of soils are summarized in Table 1.

### 2.2. Chemicals and Instruments

All chemicals and reagents, including acetic acid, sodium hydroxide, phenolphthalein indicator, sodium carbonate, chloramine-T, and sodium thiosulfate, were of ACS reagent grade or higher purity (Sinopharm Group Chemical Reagent Co., Ltd., Shanghai, China) and used without further purification. Experimental measurements employed a ultraviolet-visible spectrophotometer (Hitachi U-3010, Tokyo, Japan) for Cr(VI) quantification at 540 nm, a high-speed benchtop centrifuge (Xiangyi H-1650, Changsha, China) operated for soil suspension separation, and a analytical balance(METTLER TOLEDO ML204/02, Shanghai, China) for precise sample weighing.

### 2.3. Experimental Procedure

**Figure 2:**
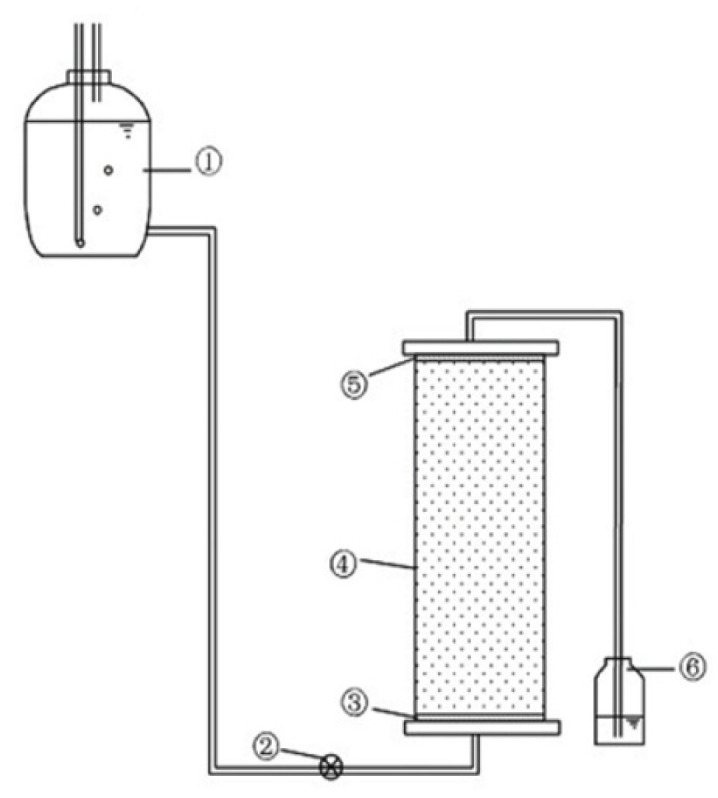
Schematic diagram of the soil column leaching experimental setup: (1) feed solution reservoir; (2) flow control valve; (3) bottom quartz sand layer with nylon mesh filter; (4) soil column; (5) top quartz sand layer with nylon mesh; (6) effluent collection bottle.

The experimental setup shown in Figure 2 consisted of a transparent plexiglass soil column (10 cm diameter × 36 cm height) equipped with stainless steel flange-sealed end caps containing 1 cm diameter influent/effluent ports, connected to a 5 L Mariotte-type constant-head device that maintained precise hydraulic control through a three-port rubber stopper assembly featuring a long vent tube extending to the bottle base and a short delivery tube, with the system first primed with deaired water before experiments by sealing the vent tube while opening the delivery tube to establish stable flow conditions while allowing visual monitoring of the wetting front progression through the column walls.

(1) Soil Column Preparation

To achieve uniform bulk density, the soil columns were packed using a layered approach. Each layer consisted of the same soil type, with carefully controlled mass increments to maintain consistency throughout the column.

(1) $$W=V\rho_{d}$$

*V* represents the volume of each soil layer, with units of [L^3^]; and *ρ*_d_ denotes the bulk density of the soil, with units of [ML^−3^].

(2) Bromide anion (Br^−^) Tracer Experiment

The dispersion coefficient was determined via bromide (Br^−^) breakthrough experiments using a 10 mg/L KBr solution. The experimental setup and hydraulic conductivity determination followed established protocols, with the Mariotte bottle solution replaced by the KBr solution. Effluent samples were collected at predetermined time intervals from the column outlet, and Br^−^ concentrations were quantified using the phenol red colorimetric method. The experiment terminated when effluent Br^−^ concentrations reached equilibrium (no further concentration changes). Breakthrough curve (BTC) was generated by correlating Br^−^ concentrations with elapsed time.

(3) Cr(VI) Migration Experiment

The Cr(VI) transport study employed an identical experimental setup, substituting the KBr solution with 20 mg/L potassium dichromate (K_2_Cr_2_O_7_) [19,20]. Effluent samples were collected at regular intervals for Cr(VI) concentration analysis. The experiment continued until effluent Cr(VI) concentrations stabilized, indicating system equilibrium. Cr(VI) breakthrough curves were constructed by plotting concentration versus time.

## 3. Results

### 3.1. Results of the Soil Column Leaching Experiment

**Figure 3:**
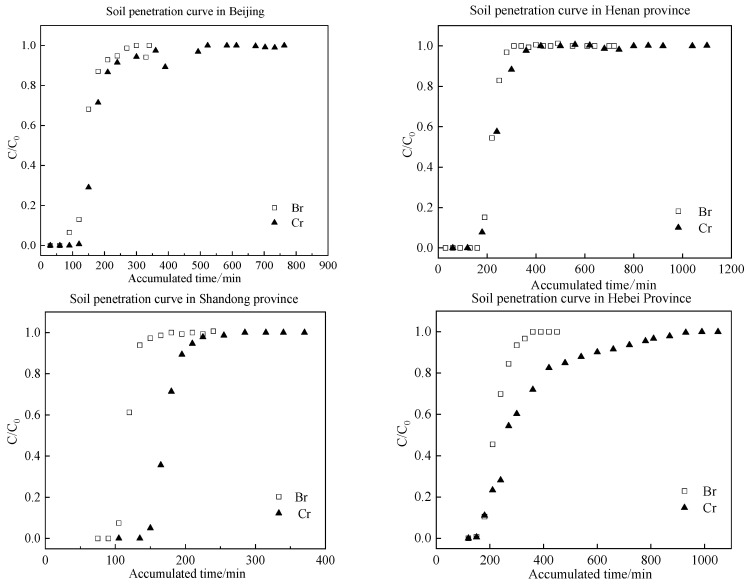

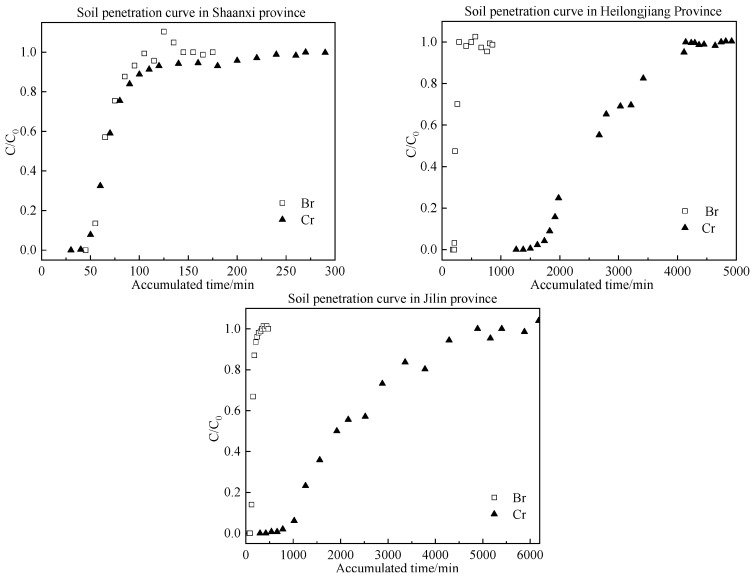
Breakthrough Curves for Br^−^ and Cr(VI).

The transport characteristics of solutes in different types of soils can be demonstrated through the use of breakthrough curves [21,22]. The breakthrough curves for a range of soil types are presented in Figure 3, with cumulative time plotted on the horizontal axis and the ratio of outflow concentration to initial concentration plotted on the vertical axis. Soil column leaching experiments were conducted using KBr as a tracer and K_2_Cr_2_O_7_ as a contaminant, resulting in the acquisition of breakthrough curves for a range of soil types, including Beijing cinnamon soil, Henan mollisol, Shandong mollisol, Hebei mollisol, Shaanxi loess, Heilongjiang mollisol, and Jilin mollisol.

As shown in Figure 2, the breakthrough curves demonstrate that Br^−^ consistently reaches equilibrium earlier than Cr(VI) in all tested soil types. This difference primarily stems from the contrasting chemical behaviors of these species: Br^−^ remain chemically inert in natural environments, showing negligible adsorption–desorption, transformation, or degradation interactions with soil matrices [23,24]. Consequently, Br^−^ migration exhibits minimal retardation effects. In stark contrast, the reactive nature of Cr(VI) leads to significant soil interactions, resulting in pronounced retardation and hysteresis phenomena. Furthermore, the time delay of Cr(VI) breakthrough relative to Br^−^ at equilibrium followed the order: Shandong mollisol < Shaanxi loess < Henan mollisol < Beijing cinnamon soil < Hebei mollisol < Heilongjiang mollisol < Jilin black mollisol. When combined with the cumulative flow consumption difference between Cr(VI) and Br^−^ at equilibrium, the retardation strength for Cr(VI) across tested soils can be preliminarily ranked as: Henan mollisol < Shandong mollisol < Beijing cinnamon soil < Shaanxi loess < Hebei mollisol < Heilongjiang mollisol < Jilin mollisol.

### 3.2. Calculation of Dispersion Coefficient (D) for Cr(VI) in Typical Soils

The dispersion coefficient (D) quantifies a porous medium’s ability to disperse solutes at a given flow velocity [25]. Macroscopically, D reflects the combined effects of groundwater velocity and pore structure characteristics on solute transport through porous media [26]. While various methods exist for determining D in soils (including formula-based calculations, horizontal column infiltration, and breakthrough curve analysis) [27], we employed the breakthrough curve method due to its superior accuracy in simulating field conditions. Specifically, we conducted Br^−^ tracer experiments in soil columns to generate breakthrough curves, from which soil-specific D values were calculated.

In the context of our calculations, the water quality model for the soil column leaching experiment is abstracted as a one-dimensional seepage and dispersion scenario, whereby Br ions with a concentration of C_0_ are continuously injected into a vertically semi-infinite soil column. It is assumed that the aquifer medium is homogeneous, with the origin of the coordinate system situated at the inlet end of the soil column. Fluid injection commences at t = 0, and the water flow is a one-dimensional flow with a constant head. The fluid is assumed to be incompressible, homogeneous, and at a constant temperature. The mathematical model for this scenario is as follows [28]:

(2) $$\left\{\begin{array}{l} \frac{\partial C}{\partial t}=D_{L}\frac{\partial^{2}y}{\partial x^{2}}-v\frac{\partial C}{\partial x}\\ C\left(x,0\right)=C_{1},x>0\\ C\left(t,0\right)=C_{0},t\geq 0\\ \underset{x\to \infty }{\lim}C\left(x,t\right)=0,t\geq 0 \end{array}\right.$$

The analytical solution obtained through Laplace transformation is as follows:

(3) $$\frac{C-C_{1}}{C_{0}-C_{1}}=\frac{1}{2}\left[erfc\left(\frac{x-vt}{2\sqrt{D_{L}t}}\right)\right]+\exp\left(\frac{vx}{D_{L}}\right)erfc\left(\frac{x+vt}{2\sqrt{D_{L}t}}\right)$$

In the case where x is of a relatively large value (vx/D > 10), the second term is found to be considerably smaller in comparison to the first term and can therefore be disregarded.

Letting $C-C_{1}=C^{\prime }$, $C_{0}-C_{1}=C_{0}^{\prime }$, we obtain

(4) $$\frac{C^{\prime }}{C_{0}^{\prime }}=\frac{1}{\sqrt{2\pi }}\int_{\frac{x-vt}{2\sqrt{D_{L}t}}}^{\infty }\exp\left(-\frac{\eta^{2}}{2}\right)d\eta$$

For a given *t*, the above equation on *x* is a normal distribution function $1-N\left[\frac{x-m}{\sigma }\right]$, its mathematical expectation m=vt, standard deviation $\sigma =\sqrt{2D_{L}t}$, and $N\left(1\right)≐0.84$, $N\left(-1\right)≐0.16$.

Following this property, for a fixed moment *t*, and assuming that the distance between the relative concentration $x_{0.84}$ at $\frac{C^{\prime }}{C_{0}^{\prime }}$ and the relative concentration $x_{0.16}$ at $\frac{C^{\prime }}{C_{0}^{\prime }}$ is the width *e* of the transition zone, $e={\left(x_{0.16}-x_{0.84}\right)}^{2}=2\sigma$.

(5) $$D_{L}=\frac{1}{8t}{\left(x_{0.16}-x_{0.84}\right)}^{2}=\frac{1}{8}{\left[\frac{x-vt_{0.16}}{\sqrt{t_{0.16}}}-\frac{x-vt_{0.84}}{\sqrt{t_{0.84}}}\right]}^{2}=\frac{v^{2}}{8t_{0.5}}{\left(t_{0.84}-t_{0.16}\right)}^{2}$$

The formula can also be converted to a formula calculated at a fixed point *x.* Let $t_{0.16}$ and $t_{0.84}$ denote the time at which the relative concentration at a point *x* reaches 0.16 and 0.84, respectively, then we have:

(6) $$D_{L}=\frac{1}{8}{\left[\frac{x-vt_{0.16}}{\sqrt{t_{0.16}}}-\frac{x-vt_{0.84}}{\sqrt{t_{0.84}}}\right]}^{2}$$

Usually, the width of the transition zone is small compared to the length of the soil column, so $\sqrt{t_{0.16}}$ and $\sqrt{t_{0.84}}$ can be approximated by $\sqrt{t_{0.5}}$, so there [29]:

(7) $$D_{L}=\frac{v^{2}}{8t_{0.5}}{\left(t_{0.84}-t_{0.16}\right)}^{2}$$

(8) $$v=\frac{L}{t_{0.5}}$$

In the aforementioned equation, the values $t_{0.16}$, $t_{0.5}$, and $t_{0.84}$ represent the respective times at which the relative concentrations at the outlet of the soil column reach 0.16, 0.5, and 0.84, respectively. The term *v* denotes the average water velocity.

The tracer experimental data for Br^−^ in typical soils, obtained through soil column leaching experiments with Br^−^, are presented in Table 2.

The dispersion coefficients for Beijing cinnamon-colored fluvo-aquic soil, Henan fluvo-aquic soil, Shandong fluvo-aquic soil, Hebei fluvo-aquic soil, Shaanxi loess soil, Heilongjiang mollisol, and Jilin mollisol were calculated using the “three-point formula” based on the tracer experimental data for bromide (Br^−^) ions. The results of the calculations are presented in Table 3. The dispersion coefficients (D) of the seven tested soils exhibited the following order: Shaanxi loess > Jilin mollisol > Beijing cinnamon-colored fluvo-aquic soil > Hebei fluvo-aquic soil > Henan fluvo-aquic soil > Shandong fluvo-aquic soil > Heilongjiang mollisol. This pattern indicates that Cr(VI) demonstrates relatively stronger longitudinal dispersion capacity in fluvo-aquic and cinnamon fluvo-aquic soils, while showing comparatively weaker dispersivity in black soils.

### 3.3. Retention Factor of Cr(VI) in Typical Soils

CXTFIT2.1, developed by the U.S. Salinity Laboratory, is a computational tool designed for simulating solute transport processes [30]. The software utilizes nonlinear least squares optimization to fit experimental breakthrough curves derived from soil column leaching experiments. Its core functionality involves solving the inverse problem of the one-dimensional convective-dispersive equation (CDE) to estimate key transport parameters, particularly the retardation factor [12]. In this study, we implemented the CXTFIT2.1 module within the STANMOD software package [31]. Our analytical approach employed (1) a deterministic equilibrium model for dispersion coefficient determination and (2) a two-region non-equilibrium model for retardation factor estimation.

The transport of solutes in soil can be influenced by physical and chemical non-equilibrium sorption processes [32]. The chemical non-equilibrium sorption model postulates that sorption occurs instantaneously at specific locations, whereas the sorption reactions at other locations are governed by a first-order reaction kinetic equation. The two-region model represents physical non-equilibrium sorption, which divides the liquid phase into mobile and immobile water regions [33].

(1) Local equilibrium assumptions

One-dimensional model for the transport of sorbing solutes in homogeneous soil under steady-state flow conditions [34].

(9) $$R\frac{\partial c}{\partial t}=D\frac{\partial^{2}c}{\partial x^{2}}-v\frac{\partial c}{\partial x}$$

(10) $$R=1+\frac{\rho K_{d}}{\theta }$$

where *c* is the concentration of solute in the liquid, [ML^−3^]; *t* is the time, [T]; *D* is the dispersion coefficients, [L^2^T^−1^]; *x* is the distance from the solute addition end, [L]; *v* is the average pore water flow rate, [LT^−1^]; *R* is the retardation factor, dimensionless; *ρ* is the dry bulk density of the soil, [ML^−3^]; *θ* is the volumetric water content, dimensionless; *K_d_* is the linear partition coefficient of the solute between the solution and adsorbed phase, [L^3^M^−1^].

(2) Non-equilibrium Hypothesis

In the calculation of the two-region model, it is assumed that the liquid phase is divided into a mobile water zone and a stagnant water zone. Convective-dispersive transport is restricted to the mobile water zone. The exchange of solutes between the mobile and stagnant water zones is constrained by the diffusion of solutes to the exchange points in the latter. This process is described using a first-order kinetic equation. The governing equations for the two-region model are as follows [34,35,36]:

(11) $$a\;=\;1,\;\left(\theta_{m}+f\rho K_{d}\right)\frac{\partial c_{m}}{\partial t}+\left[\theta_{im}+\left(1-f\right)\rho K_{d}\right]\frac{\partial c_{im}}{\partial t}=\theta_{m}D_{m}\frac{\partial^{2}c_{m}}{\partial x^{2}}-\theta_{m}v_{m}\frac{\partial c_{m}}{\partial x}$$

(12) $$\left[\theta_{im}+\left(1-f\right)\rho K_{d}\right]\frac{\partial c_{im}}{\partial t}=\alpha \left(c_{m}-c_{im}\right)$$

In Equations (11) and (12), the subscripts m and im denote the moving and non-moving water regions, f is the fraction of adsorption sites equilibrated in the moving water region, and α is the first-order mass transfer coefficient describing the rate of solute exchange between the moving and non-moving water regions.

Using dimensionless parameters, the two zone models can be reduced to the same dimensionless form:

(13) $$a\;=\;1,\;\beta R\frac{\partial c_{1}}{\partial T}=\frac{D}{vL}\frac{\partial^{2}c_{1}}{\partial Z^{2}}-\frac{\partial c_{1}}{\partial Z}-\omega \left(c_{1}-c_{2}\right)$$

(14) $$\left(1-\beta \right)R\frac{\partial c_{2}}{\partial T}=\omega \left(c_{1}-c_{2}\right)$$

In Equations (13) and (14), $T=\frac{vt}{L}$, $Z=\frac{x}{L}$, $c_{1}=\frac{c}{c_{0}}$, $c_{2}=\frac{s_{2}}{K_{d}c_{0}}$, $\beta =\frac{\theta_{m}+f\rho K_{d}}{\theta +\rho K_{d}}$, $\omega =\frac{\alpha L}{q}$, and Equation (14), β denotes the distribution of soil water in the moving and non-moving water zones, and ω denotes the ratio of the hydrodynamic residence time to the characteristic time of solute movement in the non-moving water zone.

In this study, two distinct models were employed for calculations: a deterministic equilibrium model and a non-equilibrium two-region model [12]. Firstly, for the tracer Br^−^, which were assumed to be non-reactive with the soil medium, the following steps were undertaken:

In order to estimate the dispersion coefficient (D) and the velocity (v), the deterministic equilibrium model was employed with the given value of R = 1. With a fixed value of v, the deterministic equilibrium model was then employed to estimate the dispersion coefficient (D) and the ratio of retardation (R). With both v and R held constant, the deterministic equilibrium model was utilized to estimate D.

Secondly, the following procedures were conducted for Cr(VI):

With the velocity (v) and dispersion coefficient (D) fixed at their best-fit values obtained from the bromide breakthrough curve (BTC), the deterministic equilibrium model was used to estimate R.

Similarly, with the velocity and dispersion coefficient fixed at their best-fit values from the bromide BTC, the non-equilibrium two-region model was employed to estimate R, β, and ω.

Table 4 presents the results of fitting the Br^−^ breakthrough curves using the deterministic equilibrium model (fixed R = 1). The fitted values of the dispersion coefficient (D) and velocity (v) were obtained, with the fitted v values being in close agreement with the measured results [37]. Furthermore, the correlation coefficient of the model fit was nearly 1, and the mean squared error (MSE) was approximately 0. The correlation coefficient indicates the degree of closeness between the fitted BTC and the actual BTC, with values closer to 1 indicating a more reliable fit result. The MSE represents the sum of the squares of the residuals between the fitted and measured values. A smaller MSE value indicates a superior fit performance.

Table 5 displays the results of Br^−^ breakthrough curve fitting using the deterministic equilibrium model with fixed experimentally measured velocity (v) values. The model-derived dispersion coefficients (D) and retardation factors (R) show remarkable consistency, with R values approximating unity (as expected for conservative tracers) and D values agreeing well with those in Table 4. This agreement validates the reliability of our simulation approach. The chemical inertness of Br^−^ confirms its ideal tracer characteristics for these experiments [28]. Subsequent analysis using fixed v (experimental) and R = 1 parameters yielded soil-specific D values (Table 6) that exhibited: excellent agreement between fitted and calculated values and high goodness-of-fit metrics. As the “three-point formula” calculation employs only a limited number of numerical values, whereas CXTFIT2.1 utilizes the complete experimental data set for fitting calculations, the experimental simulation values are selected as the reference values, and the fit results are deemed reliable.

Table 7 and Table 8 present Cr(VI) retardation factor (R) estimates derived from breakthrough curve (BTC) fitting using equilibrium and non-equilibrium two-region models, respectively. Both models utilized (1) experimentally determined velocities (v) and (2) Br^−^-derived dispersion coefficients (D). The non-equilibrium model additionally quantified transport mechanisms through the partition coefficient (β) and mass transfer coefficient (ω), where lower values indicate stronger non-equilibrium conditions.

**Figure 4:**
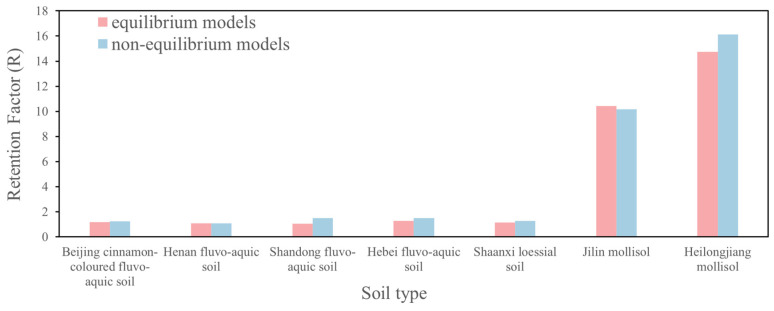
Comparison of retardation factors (R) derived from deterministic equilibrium model and two-region non-equilibrium model across seven representative soil types.

The results demonstrate that both methods yield a high degree of fit performance, with mean squared errors approaching zero and correlation coefficients close to one. The R values obtained for various soils using the two methods are comparable, but the R values estimated using the equilibrium model are slightly lower than those obtained using the non-equilibrium two-region model for Cr(VI) breakthrough curve fitting (see Figure 4 for comparative results of R values obtained from both models). This demonstrates that while the equilibrium model can achieve satisfactory fitting results under non-equilibrium conditions, it remains less accurate than the non-equilibrium model for such scenarios. These findings contrast with Wang’s [38] recommendation to use equilibrium models when β > 0.93. In this study, only Beijing cinnamon soil met this criterion (β > 0.93), while all other soils exhibited β values below this threshold. Notably, we observed an inverse relationship between retardation factor magnitude and β values, further supporting the necessity of employing non-equilibrium modeling for accurate Cr(VI) transport prediction in most soil types examined. China currently lacked established Protection of Groundwater Soil Screening Levels (PGSSLs). The retardation factor (R) was one of the core parameters for deriving PGSSLs. Accurately calculating the retardation factor enables the quantification of a pollutant’s retention capacity at the soil-groundwater interface. This facilitated a more scientifically grounded prediction of its migration pathways and eventual concentration in groundwater, thereby providing a critical basis for establishing appropriate PGSSLs tailored to diverse soil types across China.

**Figure 5:**
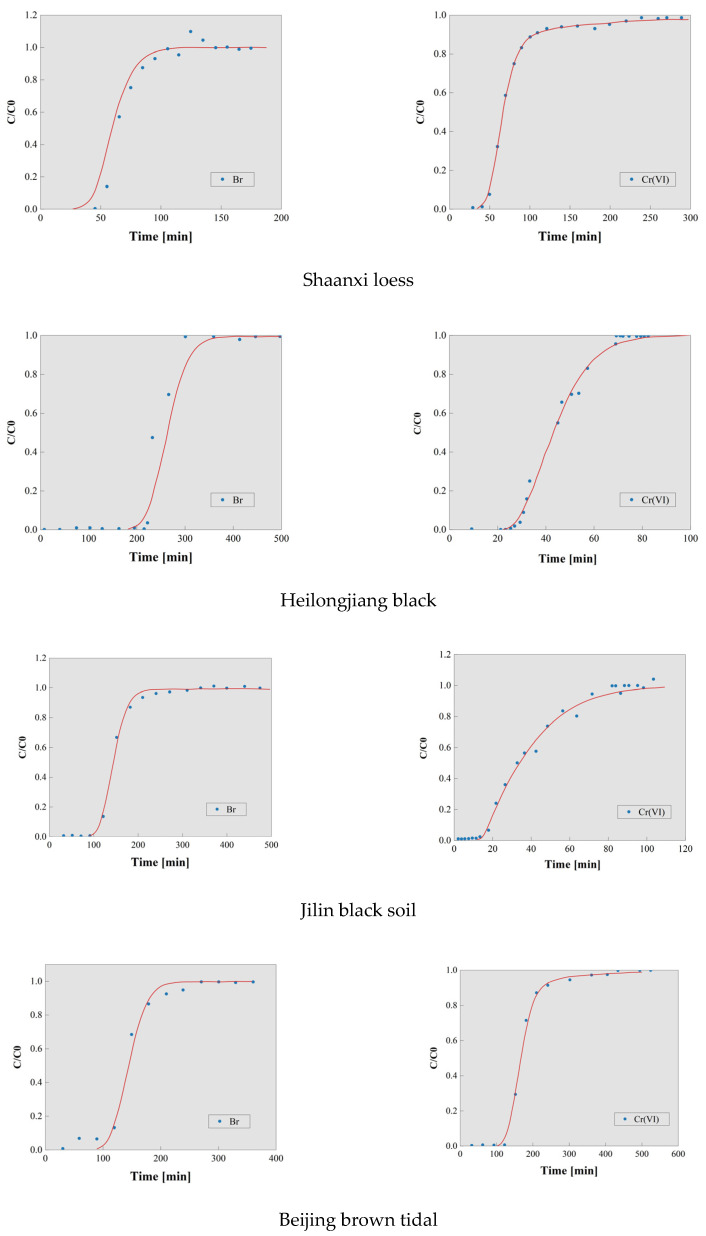

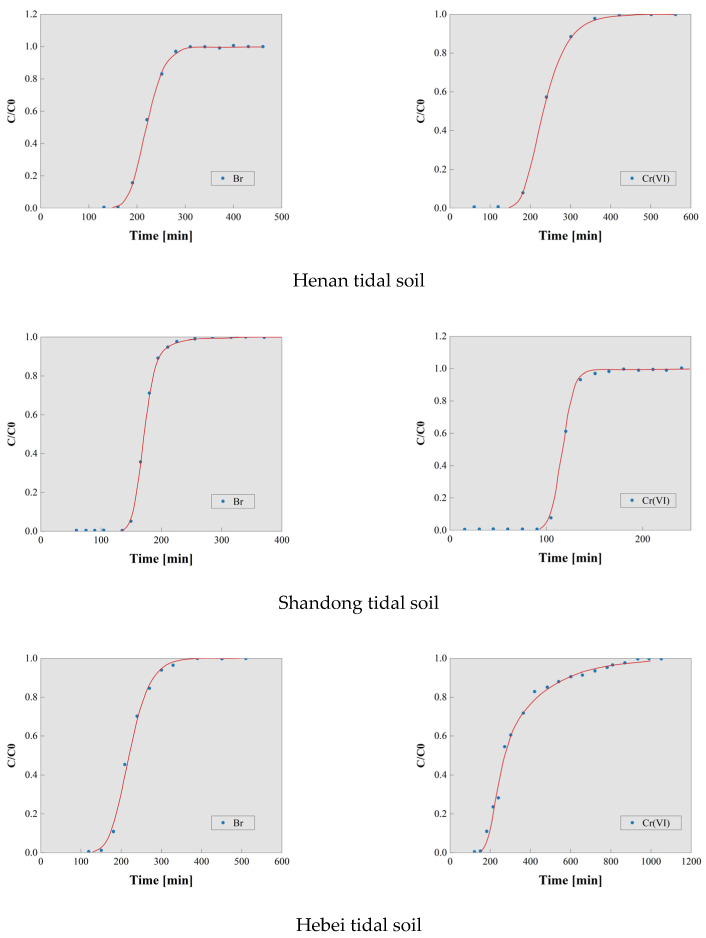
CXTFIT simulates Br (**left**) and Cr(VI) (**right**).

The results of the fitting of different soil types in this study are shown in Figure 5.

The outcomes of the fitting and calculation of the dispersion coefficient and retardation factor for diverse soil types within the present study are presented in Table 9.

A comparison of the dispersion coefficient values for different types of soils presented in Table 4 and Table 9 reveals that the calculated values are comparable to the fitted values, indicating that the fitting results obtained in this study are reasonable. Moreover, the fitting results demonstrate that the non-equilibrium two-region model is an effective approach for characterizing the transport behavior of Cr(VI) in the seven selected soil types. This indicates the existence of both mobile and immobile water regions within the soil system. In the mobile water region, convection-dispersion is the dominant process, whereas in the immobile water region, a physically non-equilibrium retardation effect is observed, resulting in stagnant water in enclosed or semi-enclosed areas within the soil system. In these regions, the primary processes are the storage and release of solutes, with solute exchange occurring via diffusion. It can therefore be concluded that the immobile water region in soil media has a significant retardation effect on solutes.

## 4. Conclusions

This study presents a novel approach by integrating Br^−^ tracer experiments with the non-equilibrium two-region model, enabling the first systematic quantification of Cr(VI) retardation factors across seven representative Chinese soil types. The findings provide significant insights into contaminant transport mechanisms, while establishing an essential theoretical framework for formulating regionally tailored soil remediation protocols. The specific conclusions are as follows:

(1) Representative soils from disparate regions of China were selected for soil column leaching experiments with the objective of obtaining breakthrough curves (BTCs) for bromide ions (as tracers) and Cr(VI) in typical soils. The data were then used to calculate the dispersion coefficients for the different soil types. The experimental data indicated that Cr(VI) exhibited relatively high longitudinal dispersion capabilities in loess and black soil, whereas its dispersion was relatively limited in cinnamon soil.

(2) Using CXTFIT2.1, retardation factors for Cr(VI) in various typical soils were calculated using both a deterministic equilibrium model and a non-equilibrium two-region model. The findings indicated that the non-equilibrium two-region model yielded more precise estimates of the retardation factors for Cr(VI) in these soils. Upon fitting the BTCs of Cr(VI) to estimate retardation factors using both the equilibrium model and the non-equilibrium two-region model, the mean squared errors for both methods were found to be close to zero, while the correlation coefficients were close to one, indicating a good fit performance. However, the retardation factors (R) calculated using the equilibrium model were found to be slightly lower than those obtained using the non-equilibrium two-region model. Nevertheless, the discrepancies between the outcomes of the two approaches were not substantial. In this study, the partition coefficient (β) values were less than 0.93 for all soils except Beijing cinnamon soil. Furthermore, soils with higher retardation factors exhibited lower β values. This indicates that, despite the equilibrium model’s continued capacity to yield satisfactory fits in non-equilibrium settings, its precision is inferior to that of the non-equilibrium model.

(3) In comparison to the traditional static adsorption batch experiments employed for the calculation of retardation factors [39], soil column leaching experiments yield results that are more closely aligned with the actual environmental conditions. The retardation factors obtained from these experiments are therefore more accurate.

## Figures and Tables

**Table 1:** Basic properties of the soils.

Soil Indices	Sand (%)	Silt (%)	Clay (%)	pH	SOM (g/kg)	CEC (cmol/kg)	Particle Density (kg/dm^3^)
Beijing cinnamon-colored fluvo-aquic soil	12.0	79.6	8.4	8.47	Not detected	7.56	2.597
Henan fluvo-aquic soil	11.0	81.5	7.5	8.01	4.78	5.37	2.682
Shandong fluvo-aquic soil	35.5	56.1	8.4	8.56	2.70	8.01	2.706
Hebei fluvo-aquic soil	5.0	74.7	20.3	8.25	13.40	15.2	2.595
Shaanxi loessial soil	2.7	85.0	12.3	6.38	43.4	30.20	2.690
Heilongjiang mollisol	8.9	78.6	12.5	8.09	12.6	12.30	2.633
Jilin mollisol	8.6	71.8	19.6	7.01	16.5	26.70	2.628

**Table 2:** Br^−^ tracer experimental data.

Soil Type	$t_{0.16}$ (min)	$t_{0.5}$ (min)	$t_{0.84}$ (min)	*v* (cm/min)
Beijing cinnamon-colored fluvo-aquic soil	121.89	139.34	175.57	0.17
Henan fluvo-aquic soil	186.31	217.77	255.44	0.12
Shandong fluvo-aquic soil	105.29	117.44	131.65	0.22
Hebei fluvo-aquic soil	181.65	216.54	270.05	0.12
Shaanxi loessial soil	56.22	59.26	79.12	0.44
Jilin mollisol	215.01	240.74	282.20	0.10
Heilongjiang mollisol	56.22	59.26	79.12	0.18

**Table 3:** Dispersion coefficients of experimental typical soils.

Soil Type	Average Pore ·Water Flowrate *v* (cm/min)	Calculated Dispersion Coefficient D (cm^2^/min)
Beijing cinnamon-colored fluvo-aquic soil	0.17	0.077
Henan fluvo-aquic soil	0.12	0.039
Shandong fluvo-aquic soil	0.22	0.034
Hebei fluvo-aquic soil	0.12	0.065
Shaanxi loessial soil	0.44	0.21
Jilin mollisol	0.18	0.12
Heilongjiang mollisol	0.10	0.027

**Table 4:** BTC estimates *D* and *v* for Br ions fitted to fixed R and equilibrium models.

Soil Type	Average Pore Water Velocity *v * (cm min^−1^)	Dispersion Coefficient *D* (cm^2^ min^−1^)	Retarding Factor *R*	Correlation Coefficient	Mean Square Error
Beijing brown tidal soil	0.17	0.063	1	0.9901	2.06 × 10^−3^
Henan tidal soil	0.12	0.029	1	0.9998	5.55 × 10^−5^
Shandong tidal soil	0.22	0.019	1	0.9990	1.63 × 10^−4^
Hebei tidal soil	0.12	0.056	1	0.9971	5.75 × 10^−4^
Shaanxi loess	0.40	0.198	1	0.9816	2.35 × 10^−3^
Jilin black soil	0.18	0.116	1	0.9953	9.42 × 10^−4^
Heilongjiang black soil	0.11	0.015	1	0.9786	5.14 × 10^−3^

**Table 5:** BTC estimates D and R for Br ions fitted to fixed *v* and equilibrium models.

Soil Type	Average Pore Water Velocity v (cm min^−1^)	Dispersion Coefficient D (cm^2^ min^−1^)	Retarding Factor R	Correlation Coefficient	Mean Square Error
Beijing brown tidal soil	0.17	0.063	1	0.9923	1.66 × 10^−3^
Henan tidal soil	0.12	0.029	1	0.9998	5.55 × 10^−5^
Shandong tidal soil	0.22	0.019	1	0.9990	1.63 × 10^−4^
Hebei tidal soil	0.12	0.056	1	0.9971	5.75 × 10^−4^
Shaanxi loess	0.44	0.217	1.1	0.9816	2.35 × 10^−3^
Jilin black soil	0.18	0.076	1	0.9968	6.97 × 10^−4^
Heilongjiang black soil	0.10	0.015	0.94	0.9786	5.14 × 10^−3^

**Table 6:** BTC estimates D for fixed v and R, equilibrium model fitting Br ions.

Soil Type	Average Pore Water Velocity v (cm min^−1^)	Dispersion Coefficient D (cm^2^ min^−1^)	Retarding Factor R	Correlation Coefficient	Mean Square Error
Beijing brown tidal soil	0.17	0.064	1	0.9915	2.02 × 10^−3^
Henan tidal soil	0.12	0.029	1	0.9996	6.57 × 10^−5^
Shandong tidal soil	0.22	0.021	1	0.9984	3.80 × 10^−4^
Hebei tidal soil	0.12	0.057	1	0.9965	6.13 × 10^−4^
Shaanxi loess	0.44	0.299	1	0.9603	7.10 × 10^−3^
Jilin black soil	0.18	0.076	1	0.9968	6.51 × 10^−4^
Heilongjiang black soil	0.10	0.022	1	0.9555	9.95 × 10^−3^

**Table 7:** Fixed v and D, BTC estimates R for equilibrium model fitting Cr(VI).

Soil Type	Average Pore Water Velocity v (cm min^−1^)	Dispersion Coefficient D (cm^2^ min^−1^)	Retarding Factor R	Correlation Coefficient	Mean Square Error
Beijing brown tidal soil	0.17	0.064	1.17	0.994847	1.02 × 10^−3^
Henan tidal soil	0.12	0.029	1.08	0.994199	1.23 × 10^−3^
Shandong tidal soil	0.22	0.021	1.06	0.943362	8.37 × 10^−3^
Hebei tidal soil	0.12	0.057	1.26	0.927071	9.36 × 10^−3^
Shaanxi loess	0.44	0.299	1.15	0.98533953	1.87 × 10^−3^
Jilin black soil	0.18	0.076	14.74	0.91042588	1.64 × 10^−2^
Heilongjiang black soil	0.10	0.022	10.41	0.957929	7.85 × 10^−3^

**Table 8:** BTC estimates R for fixed v and D, non-equilibrium models fitting Cr(VI).

Soil Type	Average Pore Water Velocity v (cm min^−1^)	Dispersion Coefficient D (cm^2^ min^−1^)	Retarding Factor R	Partition Coefficient (β)	Mass Transfer Coefficient (ω)	Correlation Coefficient	Mean Square Error
Beijing brown tidal soil	0.169745505	0.06358	1.24	0.9204	4.05 × 10^−3^	0.9984	3.75 × 10^−4^
Henan tidal soil	0.119389841	0.02939	1.09	0.8712	5.07 × 10^−2^	0.9999	5.74 × 10^−6^
Shandong tidal soil	0.22421213	0.0209	1.49	0.9765	4.82× 10^−3^	0.9998	4.64 × 10^−5^
Hebei tidal soil	0.120070107	0.05657	1.51	0.7045	2.48 × 10^−2^	0.9940	8.65 × 10^−4^
Shaanxi loess	0.438743273	0.2987	1.29	0.8502	4.50 × 10^−3^	0.9990	1.87 × 10^−4^
Jilin black soil	0.183589888	0.07611	16.13	0.4763	7.77 × 10^−2^	0.9990	1.87 × 10^−4^
Heilongjiang black soil	0.099692937	0.02228	10.17	0.6269	1.69 × 10^−1^	0.9944	1.13 × 10^−3^

**Table 9:** The dispersion coefficient and retarding factor obtained by CXTFIT fitting.

Soil Type	Dispersion Coefficient D Fitting Value	Soil Type
Beijing brown tidal soil	0.064	1.24
Henan tidal soil	0.029	1.09
Shandong tidal soil	0.021	1.49
Hebei tidal soil	0.057	1.51
Shaanxi loess	0.299	1.29
Heilongjiang black soil	0.022	10.17
Jilin black soil	0.076	16.13

## Data Availability

All processed data generated or used during the study appear in the submitted article. Raw data will be provided upon reasonable request from the corresponding author.

## References

[1] Kimbrough D.E., Cohen Y., Winer A.M., Creelman L., Mabuni C. (1999). A critical assessment of chromium in the environment. Crit. Rev. Environ. Sci. Technol..

[2] Dhal B., Thatoi H.N., Das N.N., Pandey B.D. (2013). Chemical and microbial remediation of hexavalent chromium from contaminated soil and mining/metallurgical solid waste: A review. J. Hazard. Mater..

[3] Jobby R., Jha P., Yadav A.K., Desai N. (2018). Biosorption and biotransformation of hexavalent chromium [Cr (VI)]: A comprehensive review. Chemosphere.

[4] Georgaki M.N., Charalambous M., Kazakis N., Talias M.A., Georgakis C., Papamitsou T., Mytiglaki C. (2023). Chromium in water and carcinogenic human health risk. Environments.

[5] Wang X., Li L., Yan X., Tian Y. (2020). Advances in Remediation Technologies for Chromium-Contaminated Sites. Environ. Eng..

[6] Sabur M.A., Rahman M.M., Safiullah S. (2013). Treatment of tannery effluent by locally available commercial grade lime. J. Sci. Res..

[7] Xie Y., Chen T.B., Lei M., Yang J., Guo Q.J., Song B., Zhou X.Y. (2011). Spatial distribution of soil heavy metal pollution estimated by different interpolation methods: Accuracy and uncertainty analysis. Chemosphere.

[8] Li X., Zhang J., Ma J., Liu Q., Shi T., Gong Y., Yang S., Wu Y. (2020). Status of chromium accumulation in agricultural soils across China (1989–2016). Chemosphere.

[9] Liu Y.Q., Li L., Wang Q., Cai M.L. (2009). Study on pollution situation at typical chrome residue contaminated sites and corresponding integrated remediation plan. Res. Environ. Sci..

[10] Sarangi A., Krishnan C. (2008). Comparison of in vitro Cr (VI) reduction by CFEs of chromate resistant bacteria isolated from chromate contaminated soil. Bioresour. Technol..

[11] Cederkvist K., Ingvertsen S.T., Jensen M.B., Holm P.E. (2013). Behaviour of chromium (VI) in stormwater soil infiltration systems. Appl. Geochem..

[12] Shackelford C.D., Rowe R.K., Seco e Pinto P. (1998). Contaminant transport modeling. Proceedings of the 3rd International Congress on Environmental Geotechnics.

[13] Khan A.A., Muthukrishnan M., Guha B.K. (2010). Sorption and transport modeling of hexavalent chromium on soil media. J. Hazard. Mater..

[14] Zhang Y. (2017). Study on Adsorption and Migration Characteristics of Cr(VI) in Typical Soils. Master’s Thesis.

[15] Zhao G., Wu X., Tan X., Wang X. (2011). Sorption of heavy metal ions from aqueous solutions: A review. Open Colloid Sci. J..

[16] Oluwatuyi O.E., Ojuri O.O. (2017). Environmental performance of lime–rice husk ash stabilized lateritic soil contaminated with lead or naphthalene. Geotech. Geol. Eng..

[17] Zhen Q., Zheng J., He H., Han F., Zhang X. (2016). Effects of Pisha sandstone content on solute transport in a sandy soil. Chemosphere.

[18] Schwarzenbach R.P., Gschwend P.M., Imboden D.M. (2003). Environmental Organic Chemistry.

[19] Mitchell K., Trakal L., Sillerova H., Avelar-González F.J., Guerrero-Barrera A.L., Hough R., Beesley L. (2018). Mobility of As, Cr and Cu in a contaminated grassland soil in response to diverse organic amendments; a sequential column leaching experiment. Appl. Geochem..

[20] Zhang X., Tong J., Hu B.X., Wei W. (2018). Adsorption and desorption for dynamics transport of hexavalent chromium (Cr (VI)) in soil column. Environ. Sci. Pollut. Res..

[21] Simunek J., van Genuchten M.T., Sejna M. (2008). Development and applications of the HYDRUS and STANMOD software packages and related codes. Vadose Zone J..

[22] Toride N., Leij F.J., van Genuchten M.T. (1995). The CXTFIT Code for Estimating Transport Parameters from Laboratory or Field Tracer Experiments.

[23] Ren L., Mao M. (2003). Simulation of Nonequilibrium Transport of Atrazine in Saturated Sandy Loam Soil. Acta Pedol. Sin..

[24] Wang C. (2012). Experimental Study on Vertical Transport of Cr(VI) in the Vadose Zone. Master’s Thesis.

[25] Raoof A., Hassanizadeh S.M. (2013). Saturation-dependent solute dispersivity in porous media: Pore-scale processes. Water Resour. Res..

[26] Lee J., Rolle M., Kitanidis P.K. (2018). Longitudinal dispersion coefficients for numerical modeling of groundwater solute transport in heterogeneous formations. J. Contam. Hydrol..

[27] Zhang Y., Zhang X., Xu Y., Wang X., Wang X. (2003). Research Progress on Hydrodynamic Dispersion Coefficient in Soil. Chin. J. Environ. Eng..

[28] Bai L., Wang Y., Zhang Y., Zhou Y., Yan Z., Li F. (2016). Research on the Evaluation Method of Cr (VI) Migration Parameters in Topsoil and Its Application. Soil Sediment Contam. Int. J..

[29] Wang B., Yang T., Wang B. (1985). Groundwater Pollution and Groundwater Quality Simulation Methods.

[30] Dong X., Woo H., Park H., Park J. (2013). Application of a newly developed column test device to analyze seawater transport in sandy soils. Environ. Earth Sci..

[31] Ashrafi A., van Genuchten M.T., Ghanbarian B., Ebrahimian H. (2025). Analytical solute transport modeling of furrow fertigation using the STANMOD software package. J. Hydrol. Hydromech..

[32] Nielsen D.R., Van Genuchten M.T., Biggar J.W. (1986). Water flow and solute transport processes in the unsaturated zone. Water Resour. Res..

[33] Aharoni C., Sparks D.L. (1991). Kinetics of soil chemical reactions—A theoretical treatment. Rates Soil Chem. Process..

[34] Gaber H.M., Inskeep W.P., Comfort S.D., Wraith J.M. (1995). Nonequilibrium transport of atrazine through large intact soil cores. Soil Sci. Soc. Am. J..

[35] Gamerdinger A.P., Wagenet R.J., Genuchten M.T.V. (1990). Application of two-site/two-region models for studying simultaneous nonequilibrium transport and degradation of pesticides. Soil Sci. Soc. Am. J..

[36] Genuchten M.T.V., Wagenet R.J. (1989). Two-Site/Two-Region Models for Pesticide Transport and Degradation: Theoretical Development and Analytical Solutions. Soil Sci. Soc. Am. J..

[37] Sun N.Z., Sun A. (2014). Mathematical Modeling of Groundwater Pollution.

[38] Wang D., Jiang X., He J., Bian Y., Gao H. (2007). Adsorption and Transport Characteristics of Cd^2+^ in Acidic Soils. Environ. Chem..

[39] De Souza Braz A.M., Fernandes A.R., Ferreira J.R., Alleoni L.R.F. (2013). Prediction of the distribution coefficients of metals in Amazonian soils. Ecotoxicol. Environ. Saf..

